# Involvement of GABAergic and Adrenergic Neurotransmissions on Paraventricular Nucleus of Hypothalamus in the Control of Cardiac Function

**DOI:** 10.3389/fphys.2018.00670

**Published:** 2018-06-04

**Authors:** Michelle M. Mendonça, Joice S. Santana, Kellen R. da Cruz, Danielle Ianzer, Paulo C. Ghedini, Eugene Nalivaiko, Marco A. P. Fontes, Reginaldo N. Ferreira, Gustavo R. Pedrino, Diego B. Colugnati, Carlos H. Xavier

**Affiliations:** ^1^Department of Physiological Sciences, Institute of Biological Sciences, Federal University of Goiás, Goiânia, Brazil; ^2^Neurocardiology Laboratory, School of Biomedical Sciences and Pharmacy, University of Newcastle, Callaghan, NSW, Australia; ^3^Department of Physiology and Biophysics, Institute of Biological Sciences, Federal University of Minas Gerais, Belo Horizonte, Brazil

**Keywords:** cardiac contractility, paraventricular nucleus, autonomic nervous system, cardiac function, sympathetic nervous system

## Abstract

Sympathetic premotor neurons of the paraventricular hypothalamus (PVN) play a role in hemodynamics adjustments during changes in body fluid homeostasis. However, PVN contribution to the tonic control of cardiac function remains to be systematically studied. In this study, we assessed whether GABAergic and adrenergic synapses, known for being active in the PVN, are involved in the control of cardiac function. Adult male Wistar rats (250–350 g; *n* = 27) were anesthetized with urethane (1.2–1.4 g/kg i.p.) and underwent catheterization of femoral artery to record blood pressure and heart rate. The femoral vein was used to inject the vasoactive agents phenylephrine (10 μg/kg) and sodium nitroprusside (10 μg/kg) and to supplement anesthesia. The cardiac left ventricle was catheterized to record left ventricular pressure and its derivative. Craniotomy allowed for injections (100 nL) into the PVN of: muscimol (20 mM), bicuculline methiodide (0.4 mM), propranolol (10 mM), isoproterenol (100 μM), phentolamine (13 mM), phenylephrine (30 nM). We found that: (i) inhibition of PVN by muscimol, reduced arterial pressure, cardiac chronotropy and inotropy; (ii) disinhibition of PVN neurons by bicuculline evoked positive chronotropy and inotropy, and increase blood pressure; (iii) PVN alpha adrenergic receptors control cardiac chronotropy and inotropy; (iv) beta adrenergic receptors of the PVN do not influence cardiac function; (v) afterload does not contribute to the PVN-evoked inotropy. Our results indicate that the modulation of the activity of PVN neurons exerted by GABAergic and adrenergic mechanisms contribute to the control of cardiac function.

## Introduction

Sympathetic neural outflow to blood vessels and to the heart is prominently determined by activity of sympathetic premotor neurons located in the rostral ventrolateral medulla (RVLM) and in the paraventricular hypothalamus (PVN) (Dampney et al., 2005; Guyenet, 2006). Through monosynaptic projections, neurons composing these nuclei reach preganglionic sympathetic cells in the spinal intermediolateral column (IML) (Strack et al., 1989).

Magnocellular neurons of the PVN project to the neurohypophysis and control electrolyte balance (Swanson and Sawchenko, 1983; Ambuhl et al., 1994; Blair et al., 1996; Chen and Toney, 2001). The PVN also comprises parvocellular regions that are predominantly sympathetic premotor neurons. Through direct projections that connect to several topographic levels of IML or by reaching sympathetic premotor neurons of the RVLM, the PVN controls the sympathetic outflow with pivotal influence on cardiovascular regulation (Sawchenko and Swanson, 1982a; Martin et al., 1991; Shafton et al., 1998; Daftary et al., 2000; Haywood et al., 2001).

The activity of PVN sympathetic premotor neurons is controlled by GABAergic inhibitory synapses (Decavel and Van den Pol, 1990; Martin et al., 1991; Patel et al., 2000). Considerable amount of research shows that decreases in the inhibitory activity combined with increases in excitatory inputs to the PVN results in exacerbated sympathetic activity, which occurs in chronic heart failure (Patel et al., 2000; Allen, 2002; Akine et al., 2003). Increases in sympathetic outflow to the heart (Li et al., 2006), renal and splanchnic beds, are consequence of changes in PVN activity (Carillo et al., 2012) that is found in hypertensive models (Haywood et al., 2001; Allen, 2002; Akine et al., 2003). Increases in the activity of PVN neurons are strongly correlated to increases the sympathetic outflow to the heart (Ramchandra et al., 2013). GABAergic influences upon the PVN seem to be a limiting step in the genesis of the sympathetic overactivity that composes the cardiovascular pathophysiology, since the reductions in blood pressure and sympathetic activity evoked by PVN inhibition are significantly greater in hypertensive animals (Allen, 2002; Akine et al., 2003). Conversely, disinhibition of the PVN by GABA_A_ receptor antagonists increases sympathetic outflow to different vascular beds (Reddy et al., 2005; Li et al., 2006). The PVN also participates in the modulation of baroreflex pathways, mainly in the generation of sympathetic efferent activity. Patel and Schmid (1988) detected increases in the activity of lumbar sympathetic nerves after changes in the activity of PVN neurons. They further observed that the sympathetic responses produced after baroreflex activation are modulated by the PVN (Patel and Schmid, 1988). Subsequently, it was observed that alterations in the inhibitory activity upon PVN results in increased sympathetic efferent activity to the heart, likely contributing to the physiopathology of heart failure (Li and Patel, 2003).

In addition to being controlled by GABA, catecholaminergic influences seem to be important to set the activity of PVN neurons that also expresses α- and β-adrenergic receptors (Leibowitz et al., 1982; Saphier and Feldman, 1991). Brainstem areas are the main sources of noradrenaline (NE) to the PVN (Sawchenko and Swanson, 1982b). Increases in the activity of noradrenergic inputs to the PVN could be responsible for shifting basal sympathetic tone observed in heart failure (Arabia et al., 2002). The PVN activity of non-anesthetized rats depend on β-adrenergic sensitization to reset the baroreflex control (Wang et al., 2013).

Considering the evidence for the role of PVN in sympathetic regulation of the cardiovascular system, the hypothesis we raise is that the PVN contributes to the control of cardiac function. To the best of our knowledge, the literature lacks data on the control of cardiac performance by the PVN during normotension. In this study, we sought to assess the contribution of GABAergic and adrenergic receptors in the PVN to the control of cardiac function in normotensive anesthetized animals.

## Materials and Methods

### Animals and Surgery

The animals were provided by the local animal facility (Biotério Central UFG). Experimental procedures were approved by Ethics Committee on Animal Use of the Federal University of Goiás (Brazil) (CEUA-UFG protocol 059/15). We used male Wistar rats (250–350 g). Our procedures followed the rules established by the Brazilian Committee for Animal Experiment (COBEA) and by United States National Institutes of Health Guide for the Care and Use of Laboratory Animals. All efforts were made to minimize the number of animals needed to complete the experiments.

All drugs were diluted in sterile NaCl 0.9%. Experiments were conducted under urethane anesthesia (1.4 g/kg i.p.); its adequacy was verified by the absence of a withdrawal response to a nociceptive stimulation of a hindpaw. Supplemental doses of urethane were given when necessary. Polyethylene catheters were placed into the femoral artery and vein for recordings arterial pressure (AP) and for drug injections, respectively. Left ventricular pressure (LVP) was measured using polyethylene catheter inserted into the left ventricle through the right common carotid artery. Peak values of the first derivative from LVP, i.e., LVdP/dt peak (a measure of contractility), were computed online. Subsequently, the animals were positioned on a heating pad in a prone position, and their head was placed in a stereotaxic frame (AVS Projetos, Brazil), in ventral decubitus with the head positioned -3.3 mm above the interaural line. The skull was exposed in the midline for visualization of Bregma. The location coordinates of PVN were: anteroposterior: -1.8 mm; dorso-ventral: -7.8 to -8.0 mm; and lateral: 0.7 mm. After performing a local craniotomy, which allowed for needle insertion into the rat brain, an ultra-fine tipped glass micropipette was directed to the PVN and drugs were injected. The volume for all drugs, including the Evans Blue dye, was 100 nl. Body temperature was monitored using rectal thermometer and maintained in the range of 37–37.5°C with a heating pad.

### Experimental Design

Twenty-seven rats were used in the study. Animals were anesthetized and instrumented for cardiovascular recordings and central injections, split in the four experimental series described below. After waiting about 20 min for stabilization of cardiovascular parameters, injections of drugs into the PVN were performed unilaterally. The side for injections into the PVN was randomly chosen. Injection of NaCl 0.9% was performed into the PVN of some animals as control. Timeline of the experimental sequences and injections performed in every group of animals are represented in Supplementary Figure 1.

#### Experiment 1 – Contribution of Afterload to the Cardiac Inotropic Responses Evoked From PVN/Cardiac Responses Evoked by Inhibition of PVN Neurons

In order to study the effect of afterload on the cardiac contractility (von Anrep, 1912), animals (*n* = 7) received i.v. injections of the vasoconstrictor phenylephrine (PHE 10 μg/kg) and of the vasodilator sodium nitroprusside (SNP 10μg/kg) in a randomly chosen order. Changes evoked by vasoactive drugs injections were compared to those evoked by inhibition and activation of the PVN (see “Data Acquisition and Analysis” for details) (Xavier et al., 2013). Subsequently to the last i.v. injection and after cardiovascular parameters returned to stability (about 10 min), injection (100 nL) of the GABA_A_ agonist muscimol (MUSC; 12 mM) was made into the PVN (Allen, 2002). The amplitude of the responses (maximal) evoked by muscimol (sampled within subsequent 20 min period) were compared to baseline values.

#### Experiment 2 – Cardiac Responses Evoked by Disinhibition/Activation of PVN Neurons

After cardiovascular parameters stabilized, the animals (*n* = 9) received an injection (100 nL) of the GABA_A_ antagonist bicuculline methiodide (BMI) (0.4 mM) and a 20-min period was waited to observe BMI-evoked responses. The amplitudes of the responses (maximal) evoked by injection of BMI (sampled within subsequent 20 min period) were compared to baseline values.

#### Experiment 3 – Contribution of the α-Adrenergic Receptors in the PVN to the Control of Cardiac Function

The animals (*n* = 6) received injection (100 nL) of the α-adrenergic agonist phenylephrine (PHE 30 nM) into the PVN (Tsushima and Fujimoto, 1997; Zhou et al., 2010). Following 30 min, unilateral injection (100 nL) of the α-adrenergic antagonist phentolamine (PHT 13 mM) was performed into the PVN (Zhou et al., 2010). The amplitudes of the responses (maximal) evoked by PHE and PHT (sampled within subsequent 20 min period after every injection) were compared to baseline values.

#### Experiment 4 – Contribution of β-Adrenergic Receptors in the PVN to the Control of Cardiac Function

The animals (*n* = 5) received injection (100 nL) of the β-adrenergic agonist isoproterenol (ISO 100 μM) into the PVN (Saphier, 1993; Sun et al., 2005). Following 30 min, unilateral injection (100 nL) of the β-adrenergic antagonist propranolol (PROP 10 mM) was performed (Saphier, 1993; Tsushima et al., 1994; Sun et al., 2005; Zhou et al., 2010). The amplitudes of the responses (maximal) evoked by PROP and ISO (sampled within subsequent 20 min period after every injection) were compared to baseline values.

### Histology

At the end of experiments, the animals received an injection of Evans blue dye (100 nl) in the PVN. The brains were removed, kept in paraformaldehyde (10%) and then transferred to a 30% sucrose solution for 48 h prior to sectioning cut (40 μm) in the cryostat. For histological analysis, sections were mounted on slides previously gelatinized and stained with neutral red. The injection sites were compared with the atlas of Paxinos and Watson (1986).

### Data Acquisition and Analysis

Data were acquired using PowerLab 4/20 and LabChart 7.0 (ADInstruments, Sydney, NSW, Australia) and displayed online. Statistics were performed using GraphPad Prism 6. In order to control for the afterload-induced contractility changes, we compared the AP-dependency contractility index computed from values obtained during the PVN activation with that from values obtained during phenylephrine and nitroprusside administration. This index was calculated according to the following equation: I = (ΔdP/dt peak)/(ΔMAP) where ΔMAP and ΔdP/dt peak represent difference between the basal level and the maximal values for each variable obtained after either PVN activation/inhibition or after i.v. injections of phenylephrine and SNP (Xavier et al., 2013).

Comparisons between responses evoked by microinjections into the PVN were determined by two-tailed paired student *t*-test. Significance was taken at *P* < 0.05. Data are expressed as mean ± SE.

## Results

**FIGURE 1:**
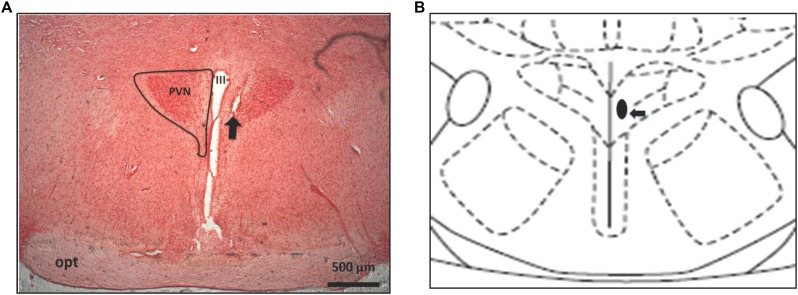
Frontal section of the rat brain (40 μm) **(A)** and schematic representation showing the center of injection into PVN **(B)**. The arrow indicates a microinjection site in PVN. PVN, paraventricular hypothalamus; III, third ventricle; OPT, optic tract.

**Figure 1** shows a histological view of a coronal section and depicts typical hypothalamic spots targeted in our experiments. The slides were compared with the diagrams of Paxinos and Watson (1986) to confirm injection sites. Supplementary Table 1 shows statistic details.

### Agonism of GABA_A_ Receptors in the PVN

**FIGURE 2:**
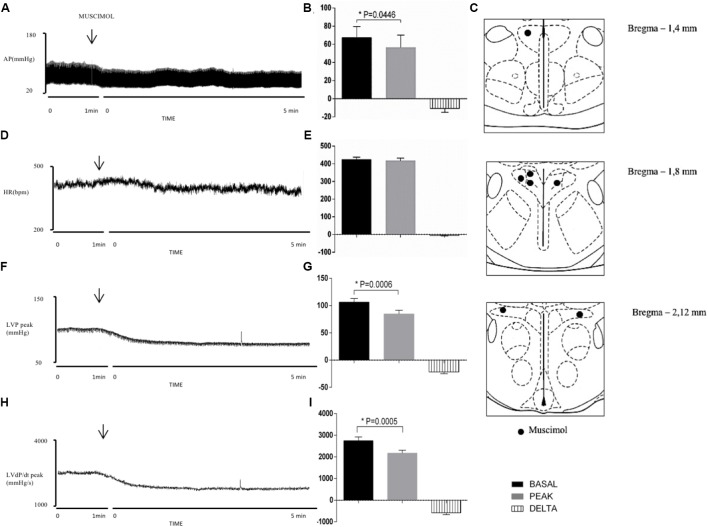
Left column – Chart records illustrating the responses of MAP **(A)**, HR **(D)**, LVP peak **(F)**, and LVdP/dt peak **(H)** produced by the unilateral microinjection of muscimol in the PVN (arrows indicate the moment of injection). Middle column – Mean maximal changes in MAP **(B)**, HR **(E)**, LVP peak **(G)**, and LVdP/dt peak **(I)** in response to unilateral microinjections of muscimol (100 nl). Values refer to the means collected in the basal period (2 min before the central injection), and at the maximum point of response to microinjection of muscimol and its variation. ^∗^*P* < 0.05. MAP, mean arterial pressure; HR, heart rate; LVP peak, maximum pressure in the left ventricle; LVdP/dt peak, first derivative of cardiac left ventricle pressure. Right column – Schematic representation of the injection sites stained by Evans blue dye from group injected with muscimol (100 nl) into PVN **(C)**. *n* = 7.

**Figure 2C** shows the distribution of injection sites of muscimol into the PVN. **Figures 2A,D,F,H** show the effects of muscimol after injection into the PVN. The maximal responses were reached following 5 min. **Figures 2B,E,G,I** demonstrate group data showing that the activation of GABA_A_ receptors in the PVN significantly changed MAP (Δ: -11 ± 4 mmHg, *P* = 0.0446 vs. basal), LVP peak (Δ: -22 ± 3 mmHg, *P* = 0.0006 vs. basal); LVdP/dt peak (Δ: -580 ± 87 mmHg/s, *P* = 0.0005 vs. basal), without provoking substantial changes in HR (Δ: -6 ± 3 bpm, *P* = 0.0791 vs. basal).

### Antagonism of GABA_A_ Receptors in the PVN

**FIGURE 3:**
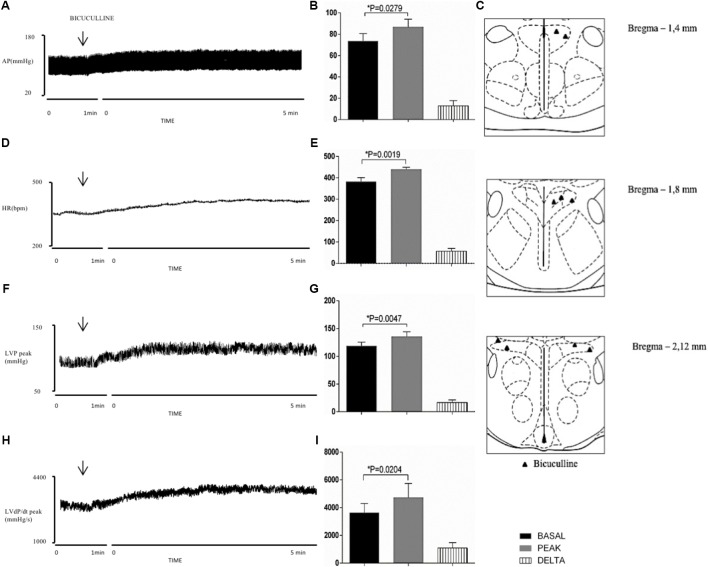
Left column – Chart records illustrating the responses of MAP **(A)**, HR **(D)**, LVP peak **(F)** and LVdP/dt peak **(H)** produced by the unilateral microinjection of bicuculline in PVN (arrows indicate the moment of injection). Middle column – Mean maximal changes in MAP **(B)**, HR **(E)**, LVP peak **(G)**, and LVdP/dt peak **(I)** in response to unilateral microinjections of bicuculline (100 nl). Values refer to the means collected in the basal period (2 min before the central injection), and at the maximum point of response to microinjection and its variation. ^∗^*P* < 0.05. MAP, mean arterial pressure; HR, heart rate; LVP peak, maximum pressure in the left ventricle; LVdP/dt peak, first derivative of cardiac left ventricle pressure. Right column – Schematic representation of the injection sites stained by Evans blue dye from group injected with bicuculline (100 nl) into PVN **(C)**. *n* = 9.

**Figure 3** shows the distribution of injection sites of BMI in the PVN (**Figure 3C**). The drug effects were evident just after injection into the PVN, and maximal responses were reached following 5 min (**Figures 3A,D,F,H**). **Figures 3B,E,G,I** demonstrate group data showing that the blockade of GABA_A_ receptors in the PVN significantly increased MAP (Δ: 13 ± 5 mmHg, *P* = 0.0279 vs. basal), HR (Δ: 57 ± 13 bpm, *P* = 0.0019 vs. basal), LVP peak (Δ: 17 ± 4 mmHg, *P* = 0.0047 vs. basal) and LVdP/dt peak (Δ: 1089 ± 388 mmHg/s, *P* = 0.0204 vs. basal).

### Contribution of α-Adrenergic Receptors in the PVN

**FIGURE 4:**
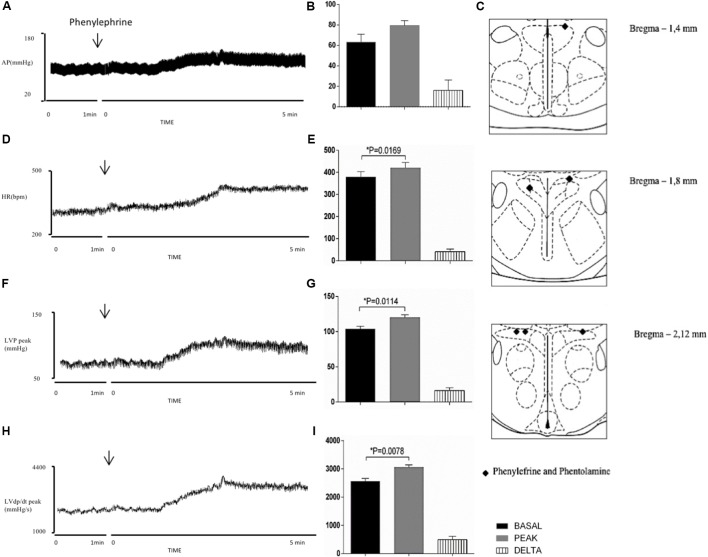
Left column – Chart records illustrating the responses of MAP **(A)**, HR **(D)**, LVP peak **(F)** and LVdP/dt peak **(H)** produced by the unilateral microinjection of phenylephrine into PVN (arrows indicate the moment of injection). Middle column – Mean maximal changes in MAP **(B)**, HR **(E)**, LVP peak **(G)**, and LVdP/dt peak **(I)** in response to unilateral microinjections of phenylephrine (100 nl). Values refer to the means collected in the basal period (2 min before the central injection), and at the maximum point of response to microinjection and its variation. ^∗^*P* < 0.05. MAP, mean arterial pressure; HR, heart rate; LVP peak, maximum pressure in the left ventricle; LVdP/dt peak, first derivative of cardiac left ventricle pressure. Right column – Schematic representation of the injection sites stained by Evans blue dye from group injected with phenylephrine and phentolamine (100 nl) group into PVN **(C)**. *n* = 6.

**FIGURE 5:**
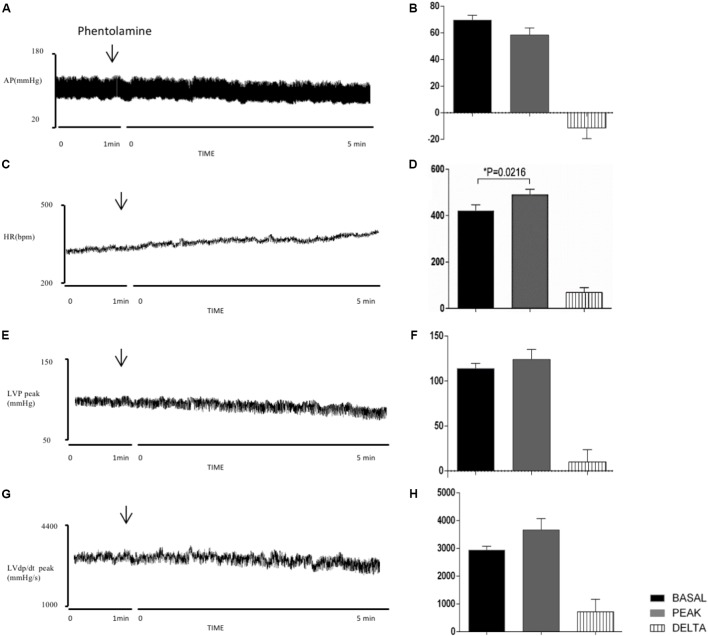
Left column – Chart records illustrating the responses of MAP **(A)**, HR **(C)**, LVP peak **(E)**, and LVdP/dt peak **(G)** produced by the unilateral microinjection of phentolamine in PVN (arrows indicate the moment of injection). Right column – Mean maximal changes in MAP **(B)**, HR **(D)**, LVP peak **(F)**, and LVdP/dt peak **(H)** in response to unilateral microinjections of phentolamine (100 nl). Values refer to the means collected in the basal period (2 min before the central injection), and at the maximum point of response to microinjection and its variation. ^∗^*P* < 0.05. MAP, mean arterial pressure; HR, heart rate; LVP peak, maximum pressure in the left ventricle; LVdP/dt peak, first derivative of cardiac left ventricle pressure. *n* = 6.

**Figure 4C** shows the sites where PHE and PHT were injected into the PVN. PHE (**Figures 4A,D,F,H**) and PHT (**Figures 5A,C,E,G**) injections into the PVN caused significant alterations in the cardiovascular parameters, evident just after injection into the PVN and reaching maximal responses following 5 min past injection.

Injection of the alpha-adrenergic agonist PHE into the PVN significantly increased HR (Δ: 41 ± 12 bpm, *P* = 0.0169 vs. basal), LVP peak (Δ: 16 ± 4 mmHg, *P* = 0.0114 vs. basal) and LVdP/dt (Δ: 496 ± 115 mmHg/s, *P* = 0.0078 vs. basal) (**Figures 4B,E,G,I**). However, there was no significant change in MAP (Δ: 16 ± 10 mmHg, *P* = 0.174 vs. basal). **Figures 5B,D,F,H** demonstrate that the injection of the alpha-adrenergic antagonist PHT into the PVN significantly changed HR (Δ: 69 ± 21 bpm; *P* = 0.0216 vs. basal). LVP peak (Δ: 10 ± 14 mmHg, *P* = 0.4917 vs. basal), LVdP/dt peak (Δ: 721 ± 447 mmHg/s, *P* = 0.1679 vs. basal) and MAP (Δ: -11 ± 8 mmHg, *P* = 0.2242 vs. basal) were unchanged.

### Contribution of β-Adrenergic Receptors in the PVN

**FIGURE 6:**
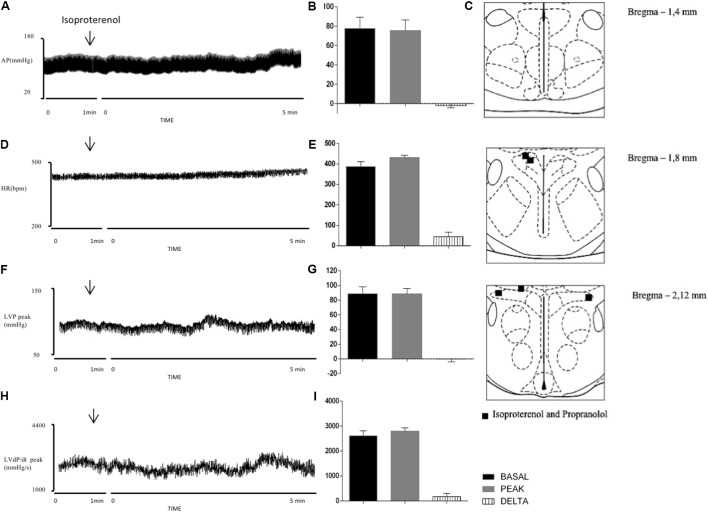
Left column – Charts records illustrating the responses of MAP **(A)**, HR **(D)**, LVP peak **(F)**, and LVdP/dt peak **(H)** produced by the unilateral microinjection of isoproterenol in PVN (arrows indicate the moment of injection). Middle column – Mean maximal changes in MAP **(B)**, HR **(E)**, LVP peak **(G)**, and LVdP/dt peak **(I)** in response to unilateral microinjections of isoproterenol (100 nl). Values refer to the means collected in the basal period (2 min before the central injection), and at the maximum point of response to microinjection and its variation. ^∗^*P* < 0.05. MAP, mean arterial pressure; HR, heart rate; LVP peak, maximum pressure in the left ventricle; LVdP/dt peak, first derivative of cardiac left ventricle pressure. Right column – Schematic representation of the injection sites stained by Evans blue dye from group injected with isoproterenol and propranolol (100 nl) into PVN **(C)**. *n* = 5.

**Figure 6C** shows the distribution of injection sites for ISO and PROP in the PVN. **Figures 6A,D,F,H** show the cardiovascular parameters before and 5 min after injections of ISO into the PVN. **Figures 6B,E,G,I** demonstrate that these injections of the beta-adrenergic agonist did not produce significant changes in MAP (Δ: -2 ± 2 mmHg, *P* = 0.415 vs. basal), HR (Δ: 44 ± 22 bpm; *P* = 0.1126 vs. basal), LVP peak (Δ: -1 ± 4 mmHg, *P* = 0.9604 vs. basal) and LVdP/dt peak (Δ: 173 ± 127 mmHg/s; *P* = 0.2469 vs. basal).

**FIGURE 7:**
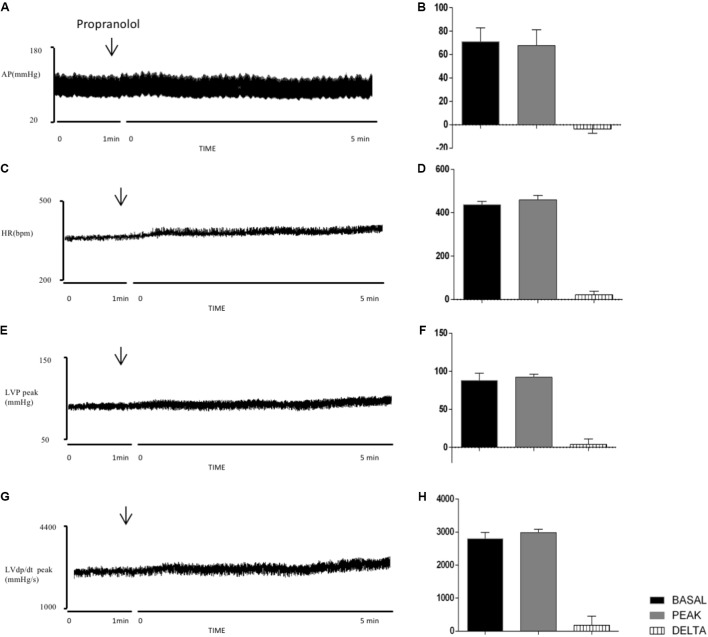
Left column – Records illustrating the responses of MAP **(A)**, HR **(C)**, LVP peak **(E)**, and LVdP/dt peak **(G)** produced by the unilateral microinjection of propranolol in PVN (arrows indicate the moment of injection). Right column – Mean maximal changes in MAP **(B)**, HR **(D)**, LVP peak **(F)**, and LVdP/dt peak **(H)** in response to unilateral microinjections of propranolol (100 nl). Values refer to the means collected in the basal period (2 min before the central injection), and at the maximum point of response to microinjection and its variation. ^∗^*P* ? 0.05. MAP, mean arterial pressure; HR, heart rate; LVP peak, maximum pressure in the left ventricle; LVdP/dt peak, first derivative of cardiac left ventricle pressure. *n* = 5.

**Figures 7A,C,E,G** show the cardiovascular parameters before and 5 min after injections of PROP into the PVN. **Figures 7B,D,F,H** demonstrate that the beta-adrenergic antagonist did not produce any significant changes in MAP (Δ: -3 ± 4 mmHg, *P* = 0.4153 vs. basal), HR (Δ: 22 ± 17 bpm, *P* = 0.2698 vs. basal), LVP peak (Δ: 4 ± 7 mmHg/s, *P* = 0.5785 vs. basal) and LVdP/dt peak (Δ: 182 ± 273 mmHg/s, *P* = 0.5407 vs. basal).

### Contribution of Afterload to the Cardiac Inotropy

**FIGURE 8:**
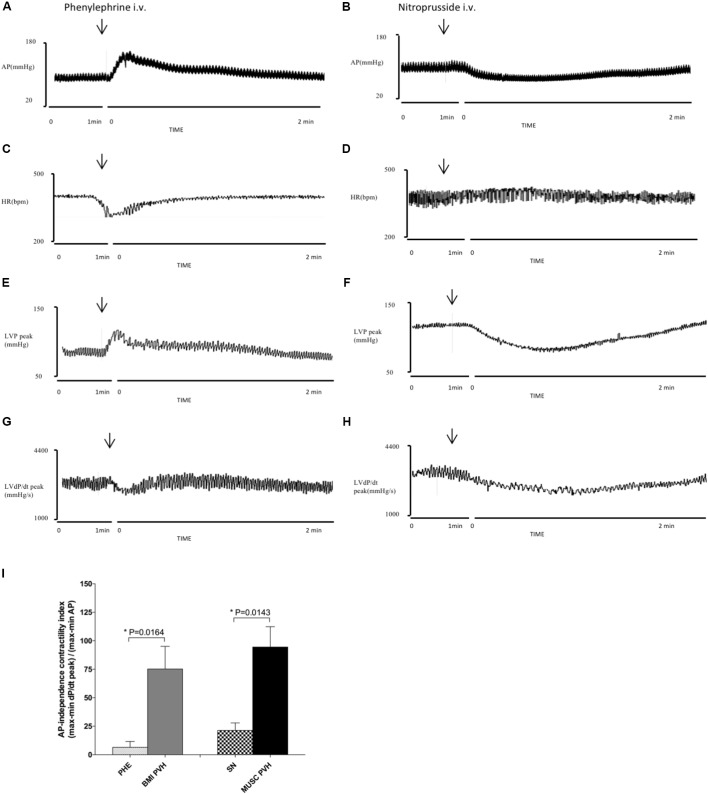
Left column – Chart records illustrating the effects of i.v. injection of phenylephrine on MAP **(A)**, HR **(C)**, LVP peak **(E)**, and LVdP/dt peak **(G)**. Right column – Records illustrates the effects of i.v. sodium nitroprusside injection on MAP **(B)**, HR **(D)**, LVP peak **(F),** and LVdP/dt peak **(H)**. Values refer to the means collected in the basal period (2 min before the central injection), and at the maximum point of response to i.v. injections. **(I)** AP-independence contractility index (for details, see “Materials and Methods”). *n* = 7.

The effects of the vasoactive drugs were evident just after intravenous injection of the PHE (**Figures 8A–D**) and SN (**Figures 8E–H**). The contractility independence index (**Figure 8I**) shows that the inotropic effects caused by microinjections into the PVN are weakly dependent on the influences of MAP, as an afterload measure. On the other hand, even though injections of vasoactive drugs produced effects on contractility, such responses were substantially lower than those observed after injections into the PVN. Therefore, it is evident that the PVN influences upon cardiac contractility are independent of its effects on blood pressure.

## Discussion

The main findings of this study are: (i) inhibition of PVN neurons by the GABA_A_ receptor agonist reduced blood pressure and cardiac inotropism; (ii) disinhibition of PVN neurons by the antagonist of GABA_A_ receptors provoked pressor, positive chronotropic and inotropic responses; (iii) α-adrenergic receptors in the PVN control cardiac chronotropism and inotropism; (iv) β-adrenergic receptors of the PVN do not influence the control of cardiac function; (v) afterload seems to exert little influence on PVN-induced inotropic responses.

Current findings indicate that the PVN contributes to the control of cardiac function. Considering that the PVN is a sympathetic premotor nucleus (Martin et al., 1991; Haywood et al., 2001), its activation or inactivation would be capable of altering cardiovascular parameters, as observed in the present study and in agreement with the results already established in the literature (Badoer, 2001; Dampney et al., 2005; Reddy et al., 2005; Gomes da Silva et al., 2012). However, the present study adds new data on the tonic control of cardiac contractile function by the PVN and the possible neurochemical mechanisms involved. So far, data on the role of PVN in the control of cardiac function at baseline conditions is scarce. Previous studies on this subject showed the participation of PVN in the cardiac autonomic control in models of arterial hypertension and heart failure (Allen, 2002; Dorn, 2010), which provides consistent evidence of the importance of PVN in the pathophysiology of cardiovascular diseases.

The PVN can modulate the activity of sympathetic outflow by relaying in premotor neurons of the RVLM and by directly reaching sympathetic preganglionic neurons of the IML column, including those spinal segments controlling the heart, kidney, adrenals, and other beds (Sawchenko and Swanson, 1982a; Strack et al., 1989; Badoer, 2001). Thus, it is likely that increases in the sympathetic outflow obtained from the PVN and from other sympathetic premotor groups modify cardiac rhythm (Narkiewicz et al., 1998), elevate circulating catecholamines and cause pressor effects (Martins-Pinge et al., 2007; Cravo et al., 2011). Recently, de Andrade et al. (2012) observed that the inhibition of PVN neurons attenuated the cardiovascular responses evoked by stress, a situation that is strongly dependent on increases in the activity of sympathetic premotor neurons. These responses were comparable to those found under blockade of cardiac sympathetic receptors with atenolol and the subsequent exposure to same stress paradigm (de Andrade et al., 2012). The predominance in the involvement of the sympathetic branch during PVN activation is further supported by another study, reporting that peripheral injection of atenolol attenuated the amplitude of the responses evoked by stimulation of PVN neurons with graded doses of NMDA (Kawabe et al., 2008). On the other hand, an attenuation in the hemodynamic responses evoked by BMI into PVN was detected following intravenous injection of the cholinergic blockers chlorisondamine and atropine, which shows a role for vagal components in the responses evoked from PVN (Martin et al., 1997).

### Methodological Considerations

Our choice was to perform experiments in anesthetized animals, since the catheterization of peripheral vessels, of cardiac ventricle and craniotomy would be logistically better and could answer the main questions raised. Literature is conflicting on the effects of urethane in physiopharmacological investigations on both central nervous system and cardiovascular system. Although urethane may attenuate cardiovascular responses to adrenergic stimuli, it appears to be the choice that minimally affects cardiovascular reflexes (Maggi and Meli, 1986a,b,c).

It has been reported the responses obtained with unilateral pharmacological interventions in the PVN are similar, regardless of the side chosen for injections (DiBona and Jones, 2001; Li and Patel, 2003; Carillo et al., 2012). Because the PVN has anatomic and functional projections to the RVLM and the latter is essential for baroreflex regulation (Badoer and Merolli, 1998), bilateral injections into the PVN could provoke more potent blood pressure responses due to the recruitment of efferent vasomotor pathways controlled by sympathetic premotor neurons of the RVLM (Allen, 2002; Akine et al., 2003). Since these ample vasomotor responses could exacerbate afterload influences in the cardiac responses evoked from the PVN, we believe that unilateral injections better met our aims, with minimal effects of hemodynamic mechanisms on cardiac function.

### Control of Cardiac Function by the GABAergic Synapses in the Paraventricular Hypothalamus

GABA is an inhibitory neurotransmitter present in the central nervous system, found in high concentrations in nuclei controlling autonomic functions (Browner et al., 1981). Changes in GABAergic tone have been associated with several changes in the control of cardiovascular system and sympathetic tone maintenance (Carillo et al., 2012). In the PVN, GABAergic nerve endings constitute approximately 50% of all synapses in this region (Decavel and Van den Pol, 1990). Thus, GABA_A_ receptors expressed in the PVN are important players in the regulation of sympathetic outflows to different vascular beds (Martin et al., 1991; Patel et al., 2000; Allen, 2002).

In order to confirm the role of GABA in the control of PVN neurons, we injected the GABA_A_ receptor agonist muscimol, which reduces cardiovascular outcomes regulated by the sympathetic branch (Allen, 2002; Akine et al., 2003; Reddy et al., 2005; Chen et al., 2010; Carillo et al., 2012). Additionally, the inhibition of PVN with the GABA_A_ receptor agonist leads to a reduction in the sympathetic nerve discharge, with a subsequent reduction in AP of normotensive and hypertensive animals. This indicates that the PVN exerts tonic influences on blood pressure and that this autonomic modulation is governed by a GABAergic tonic inhibition (Allen, 2002; Akine et al., 2003). Therefore, besides being in full agreement with the literature – where inhibition of PVN neurons by the microinjection of the GABA_A_ agonist reduces the cardiovascular parameters – we further observed a negative cardiac inotropy, as revealed by the LVP peak and LVdP/dt peak data. This allows us to suggest that the PVN also participates in the control of cardiac contractile function.

Martin et al. (1991) demonstrated that microinjections of the GABA_A_ receptor antagonist bicuculline into the PVN caused significant increases in MAP, HR and plasma catecholamines, as consequence of an overactivation of autonomic sympathetic supplies. Participation of the PVN in the modulation of this overactivity can be exerted directly through preganglionic neurons of IML or by influencing sympathetic premotor cells of RVLM (Martin et al., 1991). Later, Tagawa and Dampney (1999) observed that the pressor and sympathoexcitatory responses evoked by disinhibition of the PVN with bicuculline depend on the activation of neurons expressing angiotensin II (Ang II) receptor subtype 1 (AT1) located in the RVLM, suggesting the involvement of these receptors in the modulation of sympathetic efferent activity at the level of premotor neurons (Tagawa and Dampney, 1999). Excitatory inputs to the sympathetic premotor neurons of the RVLM also originate from the activation of PVN and are predominantly mediated by the action of the Ang II-AT1 axis (DiBona and Jones, 2001). In our study, disinhibition of PVN neurons by bicuculline injection provoked positive chronotropic and inotropic responses. Since the RVLM predominantly participates in the control of blood pressure (Irigoyen et al., 2005; Guyenet, 2006; Carillo et al., 2012), we suggest that such cardiac responses evoked from the PVN may result from direct recruitment of the PVN-IML pathway. However, the exact contribution of descending pathways from the PVN – either through IML or through RVLM – to the magnitude of our cardiac contractility findings remains to be investigated.

### Control of Cardiac Function by the Adrenergic Synapses in the Paraventricular Hypothalamus

It has been suggested an important role for adrenergic receptors in the organization of physiological responses mediated by the hypothalamus. The literature lacks reports on the role of α- and β-adrenergic receptors (Wang et al., 2013) expressed in the PVN predominantly dedicated to study neuroendocrine and cardiovascular functions, with a special focus on blood pressure (Leibowitz et al., 1982; Saphier and Feldman, 1991; Wang et al., 2013). The PVN receives projections from medullary noradrenergic neurons involved in the control of cardiovascular functions, such as the caudal ventrolateral medulla, nucleus tract solitarius, and locus coeruleus (Dampney, 1994). It has been demonstrated that NE injections into the PVN of rats evoke pressor and bradycardic responses. Harland et al. (1989) found dose-dependent increases in systolic and diastolic pressure with a concomitant negative chronotropy. Following the intravenous pretreatment with a non-selective α-adrenergic antagonist, no effect was found on bradycardia, but there was a partial inhibition of the pressure response to NE injection into the PVN (Bachelard et al., 1992), suggesting that the cardiac component elicited by NE administration into the PVN is independent of the vasomotor component.

Bachelard et al. (1991) reported that adrenaline injections into PVN increase HR, an effect partially reverted by the beta-adrenergic antagonist propranolol. Fossa et al. (2012) showed that the acute increase in NE levels in the PVN results in increases in blood pressure and HR, as consequence of an increased sympathetic activity. In addition, the microinjection of a β-adrenergic antagonist into the PVN decreased MAP of spontaneously hypertensive and Wistar-Kyoto, but not of Wistar rats (Tsushima et al., 1994). Conversely, the injection of the α-adrenergic agonist clonidine into the PVN increased MAP of Wistar rats (Hanafusa et al., 2012). Since the stimulation of PVN enhances sympathetic activity in rats and rabbits (Porter and Brody, 1985; Dampney, 1994; Deering and Coote, 2000), our findings on the effects of phenylephrine injections into the PVN are in full agreement with previous reports that showed a role for these hypothalamic α-adrenergic receptors in the autonomic control – mainly sympathetic – of cardiovascular function (Zhang and Felder, 2008). It is further supported by evidences that NE in the PVN could be responsible for the increase in basal sympathetic tone observed in cases of heart failure (Arabia et al., 2002).

The blockade of α-adrenergic receptors of the PVN by PHT produced significant chronotropic responses. It is possible that the α-adrenergic synapses of the PVN are tonically involved in the modulation of the baroreflex. In this regard, the blockade of α-adrenergic receptors may have allowed for a recruitment of pathways involved in the control of baroreflex-mediated tachycardia, since the PVN sends projections to regions composing medullary pathways of baroreflex, such as NTS and RVLM (Martin et al., 1991; Dampney, 1994; Shafton et al., 1998; Geerling et al., 2010). Hwang et al. (1998) found that α-adrenergic receptors subtype-1 of PVN are able to suppress baroreflex function. However, further studies are needed to verify whether the changes in cardiac function caused by injection of phentolamine or phenylephrine into the PVN results from changes in baroreflex gain or from isolated modifications in the activity of sympathetic premotor neurons.

We also studied the β-adrenergic receptors of the PVN and the present results, obtained in anesthetized animals, is partially in consonance with previous reports in normotensive awake rats (Tsushima et al., 1994; Wang et al., 2013). We found that β-adrenergic receptors of the PVN do not contribute to the control of cardiac function in the experimental conditions employed. In this sense, Wang et al. (2013) demonstrated an important link between β-adrenergic receptors of the PVN and the baroreflex control via PVN-RVLM projection. In that study, the intravenous injection of phenylephrine increased NE concentrations in the PVN of non-anesthetized Sprague-Dawley rats. The blockade of β-adrenergic receptors in the PVN attenuated baroreflex responses, isoprenaline (a β-adrenergic receptor agonist) in the same area improved the sensitivity of baroreflex responses. Furthermore, in the absence of baroreflex provocations, no changes in MAP or HR were observed after injection of propranolol or isoprenaline into the PVN (Wang et al., 2013). Despite being obtained in anesthetized animals, it is important mentioning that our results are in consonance with those from Wang et al. (2013). Additionally, the study of Tsushima et al. (1994) showed that the injection of the beta-2 adrenoreceptor fenoterol into PVN was able to reduce MAP of SHR and Wistar-Kyoto rats, but had no effect in Wistar rats.

### Intrinsic Mechanisms Regulating Cardiac Function

Intrinsic mechanisms contribute to adjust cardiac function, such as heart rate (Bowditch effect, treppe or frequency-dependent inotropy) and afterload (von Anrep effect) (Bowditch, 1871; von Anrep, 1912; Hallback et al., 1975; Xavier et al., 2013). Besides rate-dependency, peripheral resistance (afterload) also modifies inotropy (von Anrep, 1912; Nalivaiko et al., 2010). In our study, the influences of afterload on the contractility resulted from the ratio between contractility variation and mean AP variation [Δ(LVdP/dt peak)/Δ(MAP)] for the responses induced by administration of the vasoactive drugs. Based on the comparison between the magnitudes of contractile responses induced by the stimulation of PVN (substantial and concomitant to lower pressor responses) with those evoked by the vasoactive drugs (greatly pressor and accompanied by a discrete inotropy), we confirm that the contribution of the PVN to cardiac inotropy is regardless of afterload. This seem to be in agreement with the strong body of evidence on the involvement of PVN in the modulation of sympathetic terminals innervating the heart (Martin et al., 1991; Patel et al., 2000; Akine et al., 2003; Reddy et al., 2005; Schlenker, 2005; Kc and Dick, 2010; Ramchandra et al., 2013).

## Conclusion

We conclude that changes in the activity of PVN neurons may modify the autonomic control of cardiac function, resulting in contractile and chronotropic responses that are independent of afterload. While GABAergic synapses and alpha-adrenergic receptors of the PVN seem to contribute to the control of both chronotropy and inotropy, beta-adrenergic receptors of the PVN make minimal contribution to the control of cardiac function in anesthetized normotensive rats.

## Author Contributions

EN, MF, RF, GP, DC, and CX: conception and design. MM, JS, KdC, DI, PG, and CX: performed the experiments, analyses, and data interpretation. All authors wrote and revised the manuscript critically and approved the final version for submission and publication.

## Conflict of Interest Statement

The authors declare that the research was conducted in the absence of any commercial or financial relationships that could be construed as a potential conflict of interest.
