# Supplementary material for: Involvement of GABAergic and Adrenergic Neurotransmissions on Paraventricular Nucleus of Hypothalamus in the Control of Cardiac Function
Source: Front Physiol. 2018 Jun 4;9:670. doi: 10.3389/fphys.2018.00670 (PMC5994789; doi:10.3389/fphys.2018.00670)
Supplement: Supplementary file 1 [file Table_1.PDF]

Table 1 - statistics				
	Parameters	P value	T	df
Figure 2	MAP	0.0446	2.531	6
n=7	HR	0.0791	2.112	6
	LVP peak	0.0006	6.542	6
	LVdP/dt peak	0.0005	6.687	6
Figure 3	MAP	0.0279	2.681	8
n=9-10	HR	0.0019	4.328	9
	LVP peak	0.0047	3.735	9
	LVdP/dt peak	0.0204	2.81	9
Figure 4	MAP	0.174	1.584	5
n=6	HR	0.0169	3.52	5
	LVP peak	0.0114	3.899	5
	LVdP/dt peak	0.0078	4.294	5
Figure 5	MAP	0.2242	1.387	5
n=6	HR	0.0216	3.296	5
	LVP peak	0.4917	0.7416	5
	LVdP/dt peak	0.1678	1.612	5
Figure 6	MAP	0.415	0.9085	4
n=5	HR	0.1126	2.027	4
	LVP peak	0.9604	0.05282	4
	LVdP/dt peak	0.2469	1.355	4
Figure 7	MAP	0.4153	0.9078	4
n=5	HR	0.2698	1.28	4
	LVP peak	0.5785	0.6039	4
	LVdP/dt peak	0.5407	0.668	4
Figure 8 Musc vs. SN n=7	(peak-min dP/dt) / (peak-min AP)	0.0143	3.413	6
Figure 8 PHE vs. BMI	(peak-min dP/dt) / (peak-min AP)	0.0164	3.302	6

Table 1. Statistic details from MAP (mean arterial pressure), HR (heart rate), LVP peak (left ventricular pressure), LVdP/dt peak (a measure of contractility), of experimental groups. T (values for a 95% confidence intervals); df (degrees of freedom). Muscimol (Musc), SN (sodium nitroprusside), PHE (phenylephrine), BMI (bicuculline).
