# Supplementary figures and images for: Involvement of GABAergic and Adrenergic Neurotransmissions on Paraventricular Nucleus of Hypothalamus in the Control of Cardiac Function
Source: Front Physiol. 2018 Jun 4;9:670. doi: 10.3389/fphys.2018.00670 (PMC5994789; doi:10.3389/fphys.2018.00670)

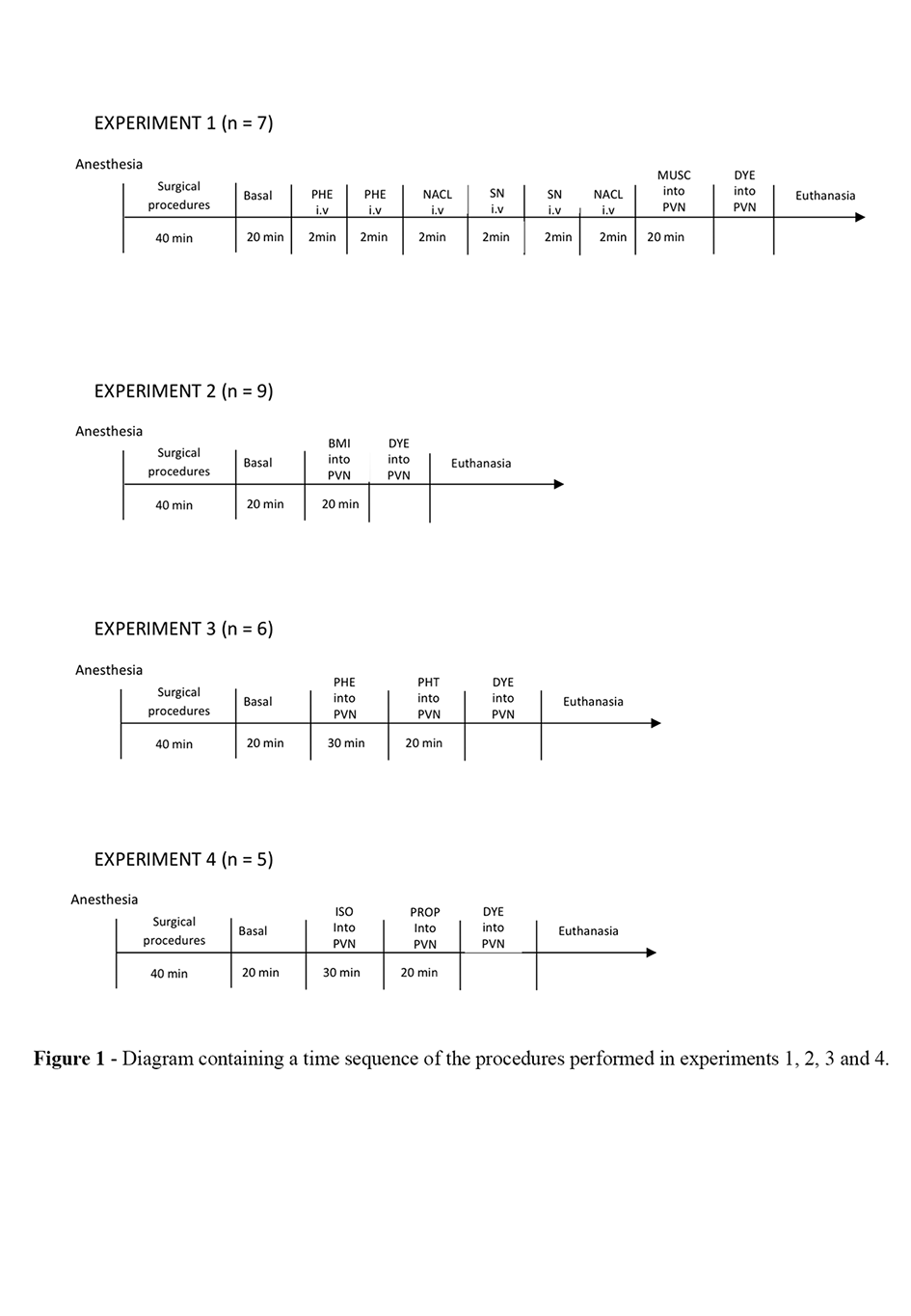

Supplement: Supplementary file 2 [file Image_1.tiff]
